# Unexpected low frequency of respiratory symptoms in an HIV-positive urban sub-Saharan population compared to an HIV-negative control group

**DOI:** 10.4102/sajhivmed.v20i1.1010

**Published:** 2019-09-26

**Authors:** Maren Kummerow, Erica J. Shaddock, Kerstin Klipstein-Grobusch, Roos B. Barth, Diederick E. Grobbee, Francois D.F. Venter, Charles Feldman, Alinda Vos

**Affiliations:** 1 Julius Global Health, Julius Center for Health Sciences and Primary Care, University Medical Center Utrecht, Utrecht University, Utrecht, the Netherlands; 2 Division of Pulmonology, Department of Internal Medicine, Charlotte Maxeke Johannesburg Academic Hospital, Johannesburg, South Africa; 3 Faculty of Health Sciences, University of the Witwatersrand, Johannesburg, South Africa; 4 Division of Epidemiology and Biostatistics, School of Public Health, Faculty of Health Sciences, University of the Witwatersrand, Johannesburg, South Africa; 5 Department of Internal Medicine and Infectious Diseases, University Medical Center Utrecht, Utrecht University, Utrecht, the Netherlands; 6 Wits Reproductive Health and HIV Institute, Faculty of Health Sciences, University of the Witwatersrand, Johannesburg, South Africa; 7 Department of Internal Medicine, Faculty of Health Sciences, University the of Witwatersrand, Johannesburg, South Africa

**Keywords:** respiratory complaints, cough, HIV, ART, sub-saharan Africa

## Abstract

**Background:**

Chronic respiratory illnesses and respiratory infections are common in HIV-positive populations. It seems reasonable that HIV-positive people experience more respiratory symptoms, such as coughing and breathlessness, than those who are HIV-negative.

**Objectives:**

This study aims to determine the frequency of respiratory symptoms in an urban African HIV-positive population.

**Method:**

A cross-sectional study was conducted in Johannesburg, South Africa, in 2016–2017. Four groups of participants were included: HIV-positive participants (1) not yet on antiretroviral therapy (ART), (2) on first-line ART, (3) on second-line ART and (4) age- and sex-matched HIV-negative controls. Data were collected on socio-demographics, respiratory risk factors and respiratory symptoms. A logistic regression analysis was performed to determine if respiratory symptoms differed between groups and to identify determinants associated with symptoms.

**Results:**

Overall, 547 participants were included, of whom 62% were women, with a median age of 37 years. Of these patients, 63% (347) were HIV-positive, 26% were ART-naïve, 24% were on first-line ART and 50% were on second-line ART. Cough and/or productive cough was reported by 27 (5%), wheezing by 9 (2%) and breathlessness by 118 (22%) of the participants. The frequency of these symptoms did not differ by HIV status after adjustment for age and sex. Breathlessness was associated with age, female sex, obesity, a history of respiratory infection and a history of airway hyper-reactivity.

**Conclusion:**

The frequency of respiratory symptoms was low in our study population except for breathlessness. HIV-positive participants, whether or not on ART, did not experience more symptoms than HIV-negative participants.

## Introduction

In the last two decades, the introduction of antiretroviral therapy (ART) has substantially improved life expectancy of HIV-positive patients. Patients who are adequately treated with ART have a near-normal lifespan compared to the general population and, as a consequence, HIV has become a chronic disease.^1^ One of the reasons for the increase in life expectancy in HIV-positive patients on ART is the lower frequency of severe and life-threatening opportunistic lung infections, such as tuberculosis and bacterial pneumonia.^2^

However, non-communicable chronic respiratory illnesses, such as chronic obstructive pulmonary disease (COPD), are more frequently seen in people living with HIV (PLHIV) compared to people without HIV.^3^ Studies have shown that PLHIV report more respiratory symptoms, such as coughing, productive cough and shortness of breath.^4^ This is of interest when considering that PLHIV are ageing and respiratory symptoms are more frequent in an older population.^5^ However, most of the literature related to respiratory symptoms in HIV infection emanate from North America. There are insufficient data on the extent of this problem in low- and middle-income countries (LMIC) where the majority of PLHIV reside and where the burden of tuberculosis and bacterial pneumonia is higher than in high-income countries (HIC) like North America.

We studied the frequency of respiratory symptoms in PLHIV whether or not on ART in an urban area in South Africa in comparison to an HIV-negative control group, as well as the determinants of respiratory symptoms.

## Methods

We conducted a cross-sectional study in Johannesburg, South Africa, from July 2016 to November 2017. We recruited four groups of participants from the Johannesburg area: HIV-positive participants not yet on ART, HIV-positive participants on first-line ART, HIV-positive participants on second-line treatment and HIV-negative control participants. The HIV-positive participants were recruited from past or ongoing randomised controlled trials (RCTs) comparing different ART regimens in a governmental HIV care facility in central Johannesburg.^6,7^ The control group was recruited by HIV-positive participants who invited their family or friends with a negative or unknown HIV status with the same age range (+/−5 years) and sex to participate in the study. All control participants underwent HIV counselling and testing according to the South African Department of Health guidelines.^8^ If a control participant tested HIV-positive and there was no history of ART use, they were counted in the first group (HIV-positive, ART-naïve) and referred to a local clinic to initiate ART. If they were on ART already, they were included in the group on first- or second-line ART, depending on their current ART regimen.

## Data collection

Data were collected during a single visit. Information on demographics and smoking was assessed with a modified version of the WHO STEPs instrument^9^. Information on medical history, respiratory symptoms, working and living circumstances and occupational exposure to potentially harmful agents was obtained using following questionnaires: The British Medical Research Council (MRC) Respiratory Questionnaire,^10,11^ the MRC dyspnoea scale,^12^ the World Health Survey,^13^ the ATS-DLD-78-A^14^ and questions used in other publications^15,16^. Respiratory symptoms evaluated in this study were ‘cough’, ‘bringing up phlegm’, ‘breathlessness’ and ‘wheezing or whistling’ according to the MRC Respiratory Questionnaire and the ATS-DLD-78. A questionnaire also included ‘cough’ and ‘bringing up phlegm’ as symptoms which were evaluated separately (Table 1).

**TABLE 1:** Questions to assess respiratory symptoms.

Symptom	Question
Cough:	Do you cough several times on most days? Yes or no
Productive cough:	Do you bring up phlegm or mucus on most days? Yes or no
Breathlessness: (≥ 2, MRC dyspnoea scale)	Which of the following statements best describes your situation? Not troubled by breathlessness except on strenuous exercise Short of breath when hurrying on the level or walking up a slight hill Walks slower than most people on the level, stops after 1.5 km or so, or stops after minutes of walking at own pace Stops for breath after walking about 100 m or after walking a few minutes in level ground Too breathless to leave house, or breathless when undressing
Wheezing or whistling:	Have you had attacks of wheezing or whistling in your chest at any time in the last 12 months? Yes or no

*Source*: Adapted from The British Medical Research Council (MRC) Respiratory Questionnaire,^10,11^ the MRC dyspnoea scale,^12^ the World Health Survey,^13^ the ATS-DLD-78-A^14^ and questions used in other publications^15,16^

MRC, Medical Research Council.

A physical examination was performed, which included measurements of height and weight. Blood sample was collected for measurement of HIV viral load and CD4-cell count (HIV-positive participants only). For participants who were recruited from one of the RCTs, laboratory data were retrieved from the RCT visit closest to our study visit.

## Data analysis

Outcomes were described as median with interquartile range for continuous variables (all non-normally distributed) and count with percentage for categorical variables. Differences in continuous variables across the four groups were tested using a Mann–Whitney *U* test and categorical variables using a Fisher’s exact test. The frequency of respiratory symptoms across the four groups was displayed in bar charts.

We combined the four respiratory symptoms in a composite outcome ‘any respiratory symptom’ that was defined as the occurrence of at least one of the respiratory complaints, namely coughing, bringing up phlegm, shortness of breath and/or wheezing or whistling. We first analysed if the frequency of respiratory symptoms differed according to HIV or ART status in three logistic regression models using ‘any respiratory symptom’ as outcome. In the first model, we assessed the unadjusted association between HIV and ART status and the occurrence of any respiratory symptom using the HIV-negative group as the reference group. The second model was adjusted for sex and age, and the third model additionally adjusted for body mass index (BMI), ever smoking, passive smoking, respiratory infections in the past (pneumonia and/or tuberculosis) and bronchial hyper-reactivity. To investigate the influence of HIV- and ART-related characteristics, we repeated the models described above including the HIV-positive participants only and using the ART-naïve group as the reference group. In the third model, HIV viral load and CD4+ cell counts were added.

Finally, we analysed which determinants were associated with any respiratory symptom. The following factors were considered in univariable analysis: HIV status, age, sex, BMI, ever smoking and passive smoking, respiratory illnesses in the past such as tuberculosis and pneumonia, history of bronchial hyper-reactivity and environmental factors (worked in the mining industry or worked in a dusty job or exposure to gas, chemical fumes or pesticides in work). All factors with a *p*-value of < 0.2 in univariate analysis as well as age and sex were then included in multivariable analysis using forced entry. A *p*-value < 0.05 was considered to be statistically significant.

For the statistical analyses, we used the statistic programme ‘IBM SPSS statistics’ version 24.0 (IBM SPSS Statistics for Windows, Version 24.0. IBM Corp., Armonk, NY).

### Ethical considerations

Ethical permission was obtained from the Human Research Ethics Committee of the University of the Witwatersrand (HREC number M160131). All participants provided written informed consent prior to participation.

## Results

Of the 548 participants, 547 were included in the study. One participant was excluded as there was no information on HIV status. Almost all participants were black Africans (99.6%), 341 (62%) were women, 394 (72%) were HIV-positive, and the median age was 37 years (Table 2). Of the HIV-positive group, 103 (26%) were ART-naïve, 94 (24%) were on first-line treatment and 197 (50%) were on second-line treatment (Table 2).

**TABLE 2:** Characteristics of the study population.

Variable	HIV-negative			HIV-positive								
				ART-naive			First-line ART			Second-line ART		
	*n*	%	median (IQR)	*n*	%	median (IQR)	*n*	%	median (IQR)	*n*	%	median (IQR)
Total(*n* = 547)	153	28.0	-	103	18.8	-	94	17.2	-	197	36.0	-
**Patient characteristics**												
Female sex	75	49.0	-	64	62.1	-	59	62.8	-	143	72.6	-
Age in years	-	-	32.0 (27.0–40.5)	-	-	33.0 (28.0–39.0)	-	-	35.0 (32.0–41.0)	-	-	42.0 (38.0-48.0)
BMI (kg/m^2^)*	-	-	24.5 (21.2–28.85)	-	-	23.5 (20.9–27.1)	-	-	24.2 (21.3–29.1)	-	-	26.3 (22.9–32.0)
Underweight	8	5.2	-	7	6.8	-	3	3.2	-	-	1.5	-
Normal weight	75	49.0	-	57	55.3	-	49	52.1	-	-	39.3	-
Overweight	36	23.5	-	24	23.3	-	20	21.3	-	50	25.5	-
Obese	34	22.2	-	15	14.6	-	22	23.4	-	66	33.7	-
Black African	151	98.7	-	103	100.0	-	94	100.0	-	197	10.0	-
**Education**												
Primary school or less	12	7.8	-	13	12.6	-	10	10.6	-	27	13.7	-
Secondary school completed	74	48.4	-	51	49.5	-	51	54.3	-	96	48.73	-
Matrix completed	37	24.2	-	29	28.2	-	24	25.5	-	59	30.0	-
College or university	29	19.0	-	9	8.7	-	8	8.5	-	13	6.6	-
**Employment**												
Employed	60	40	-	56	54.9	-	72	78.3	-	139	70.9	-
Unemployed	90	60	-	46	45.1	-	20	21.7	-	57	29.1	-
**Occupational exposure**												
Worked in the mining industry ≥ 1 year	0	0.0	-	1	1.0	-	0	0.0	-	2	1.0	-
Worked in a dusty job ≥ 1 year	2	1.3	-	1	1.0	-	7	7.6	-	6	3.1	-
Exposure to gas, chemical fumes or pesticides in work ≥ 1 year	2	1.4	-	0	0.0	-	6	6.4	-	3	1.6	-
**Respiratory illnesses**												
Pneumonia in the past	4	2.6	-	2	1.9	-	2	2.2	-	27	13.8	-
Asthma	7	4.6	-	3	2.9	-	3	3.2	-	18	9.4	-
Seasonal allergy	0	0.0	-	0	0.0	-	3	3.2	-	9	4.8	-
Active TB	1	0.7	-	0	0.0	-	0	0.0	-	7	3.6	-
TB in the past	5	3.3	-	9	8.8	-	19	20.2	-	82	41.8	-
Unknown TB status	1	0.7	-	0	0.0	-	0	0.0	-	2	1.0	-
**Smoking**												
Current daily smoker	56	36.8	-	27	26.2	-	15	16.0	-	18	-	-
Pack-years	-	-	3.7 (1.3–8.1)	-	-	3.7 (1.8–6.3)	-	-	1.8 (1.2–7.3)	-	-	7.5 (3.1–10.7)
Former smoker	6	3.9	-	7	6.8	-	8	8.5	-	14	7.1	-
Never smoker	89	58.6	-	69	67.0	-	71	75.5	-	165	83.8	-
Daily smoker of marijuana	14	9.2	-	4	3.9	-	4	4.3	-	1	0.5	-
Passive smoker	52	34.0	-	24	23.3	-	15	16.9	-	24	12.2	-
**HIV characteristics**												
Time since HIV diagnosis in months	-	-	-	-	-	0.0 (0.0–1.3)	-	-	47.0 (37.8–72.3)	-	-	108.0 (84.0–149.0)
CD4-cell count/uL	-	-	-	-	-	281.0 (191.0–400.8)	-	-	413.5 (278.5–574.5)	-	-	619.0 (429.5–798.0)
Viral load < 40 cp/mL	-	-	-	10	10.0	-	81	91.0	-	175	92.6	-
**HIV medication**												
On ART	-	-	-	66	64.1	-	94	100.0	-	197	100.0	-
Duration ART < 6 weeks	-	-	-	65	98.5	-	2	2.2	-	0	0.0	-

ART, antiretroviral therapy; IQR, interquartile range; TB, tuberculosis.

The HIV-positive group on second-line treatment had a higher percentage of women (73%, *p* < 0.001) and was older compared to the other groups (42 years, *p* < 0.001). The control group and the ART-naïve group had a high percentage of current daily smokers (37% and 26%, respectively). Thirty-four per cent HIV-positive participants reported a history of a respiratory infection such as tuberculosis or pneumonia. The numbers were especially high in the group on second-line treatment (51%).

Occupational exposure was uncommon, with only 26 participants (5%) reporting any exposure. Almost all participants used gas or electricity for cooking (99.5%) and heating (99%).

**FIGURE 1:**
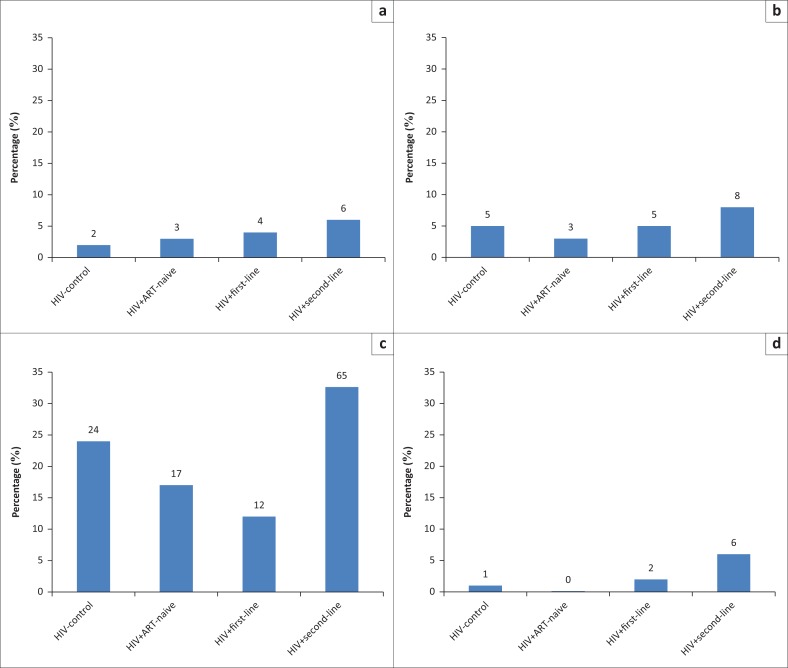
Frequency of respiratory symptoms by HIV status, (a) cough ≥ 2 weeks (*n*), (b) bringing up phlegm (*n*), (c) Medical Research Council dyspnoea scale ≥ 2 (*n*), wheezing or whistling (*n*).

Cough was reported by only six (1%) participants. Bringing up phlegm was reported by 12 (2%) participants and 9 (2%) participants reported both. Wheezing occurred in 9 (2%) participants and breathlessness in 118 (22%) participants (Figure 1).

In total, 143 participants (26%) reported at least one respiratory symptom of which 128 participants (90%) reported one symptom, 11 (8%) reported two symptoms, 3 (2%) reported three symptoms and only one participant reported all symptoms.

In the unadjusted comparison across the four groups, the HIV-positive group on second-line ART had significantly more respiratory symptoms compared to the HIV-negative group (odds ratio [OR] 2.5, 95% confidence interval [CI] 1.5–4.1) (Table 3). However, after adjustment for sex and age, there was no difference in the frequency of respiratory symptoms related to HIV or ART status. Further adjustment for respiratory risk factors did not change this finding.

**TABLE 3:** Any respiratory symptom for all participants.

Model	HIV-negative	HIV-positive					
		ART-naïve		First-line ART		Second-line ART	
		OR	95% CI	OR	95% CI	OR	95% CI
Model 1: HIV	REF	1.11	0.60–2.07	0.84	0.43–1.64	2.52	1.54–4.12*
Model 2: HIV + age + sex	REF	1.01	0.53–1.93	0.70	0.35–1.40	1.55	0.91–2.64
Model 3: HIV + age + sex + other factors†	REF	1.19	0.59–2.40	0.80	0.39–1.68	1.32	0.70–2.48

ART, antiretroviral therapy; OR, odds ratio; CI, confidence interval; REF, reference group.

† , Other factors: body mass index, ever smoking, passive smoking, respiratory infections in the past (pneumonia and/or tuberculosis), bronchial hyper-reactivity.

* , *p* < 0.05, 1.

When we restricted the analysis to the HIV-positive participants, the same crude trend was observed with participants on second-line ART having more respiratory symptoms than the other participants in the unadjusted analysis. This result disappeared after adjustment for sex and age. Additional adjustment for CD4-cell count and viral load did not change the findings.

The occurrence of any respiratory complaint was associated with age, female sex, BMI and a history of bronchial hyper-reactivity (Table 4). When limiting the analysis to breathlessness, age, female sex, BMI, bronchial hyper-reactivity and a history of pulmonary infection were the associating factors (Table 5).

**TABLE 4:** Factors associated with any respiratory symptom.

Variable	Univariable OR	95% CI	*p*	Multivariable OR	95% CI	*p*
HIV-positive	1.65	1.05–2.60	0.03	1.13 (0.65–1.96)	0.65–1.96	0.66
Age (per 5 years increase)	1.26	1.13–1.39	< 0.01	1.20 (1.06–1.35)	1.06–1.35	< 0.01*
Female sex	3.49	2.20–5.54	< 0.01	2.40 (1.38–4.17)	1.38–4.17	< 0.01*
BMI	1.11	1.07–1.14	< 0.01	1.07 (1.04–1.11)	1.04–1.11	< 0.01*
Ever smoking	0.50	0.31–0.80	< 0.01	1.05 (0.60–1.83)	0.60–1.83	0.88
Passive smoking	0.95	0.59–1.52	0.82	-	-	-
Respiratory infection in the past†	1.76	1.16–2.66	< 0.01	1.36 (0.84–2.22)	0.84–2.22	0.22
Bronchial hyper-reactivity	3.70	1.92–7.13	< 0.01	2.25 (1.11–4.57)	1.11–4.57	0.03*
Environmental exposure‡	1.52	0.66–3.50	0.32	-		-

BMI, body mass index; CI, confidence interval; OR, odds ratio.

† , Defined as either a history of pneumonia or TB.

‡ , defined as work in the mining industry or in a dusty job for > 1 year or any exposure to gas, chemical fumes or pesticides in work ≥ 1 year.

* , statistically significant at *p* < 0.05.

**TABLE 5:** Factors associated with breathlessness.

Variable	Univariable OR	95% CI	*p*	Multivariable OR	95% CI	*p*
HIV-positive	1.68 (1.03–2.76)	1.03–2.76)	0.04	1.01 (0.54–1.89)	0.54–1.89	0.98
Age (per 5 years increase)	1.34 (1.20–1.50)	1.20–1.50	< 0.01	1.34 (1.17–1.55)	1.17–1.55	< 0.01*
Female sex	5.17 (2.95–9.04)	2.95–9.04	< 0.01	3.59 (1.82–7.07)	1.82–7.07	< 0.01*
BMI	1.12 (1.09–1.16)	1.09–1.16	< 0.01	1.08 (1.04–1.12)	1.04–1.12	< 0.01*
Ever smoking	0.31 (0.18–0.56)	0.18–0.56	< 0.01	0.67 (0.34–1.32)	0.34–1.32	0.25
Passive smoking	0.84 (0.50–1.40)	0.50–1.40	0.50	-	-	-
Respiratory infection in the past†	2.13 (1.38–3.29)	1.38–3.29	< 0.01	1.76 (1.04–3.00)	1.04–3.00	0.04*
Bronchial hyper-reactivity	4.07 (2.10–7.89)	2.10–7.89	< 0.01	2.15 (1.02–4.51)	1.02–4.51	0.04*
Environmental exposure‡	0.84 (0.31–2.27)	0.31–2.27	0.73	-	-	-

BMI, body mass index; CI, confidence interval; OR, odds ratio.

† , Defined as either a history of pneumonia or TB.

‡ , defined as work in the mining industry or in a dusty job for > 1 year or any exposure to gas, chemical fumes or pesticides in work ≥ 1 year.

* , statistically significant at *p* < 0.05.

## Discussion

The frequency of respiratory complaints in our study was surprisingly low when compared to what has been reported in the literature in studies from both HIC and LMIC. For coughing, a frequency of 17%^17^ – 40%^18^ has been reported in HIC and 7% – 48%^19,20,21,22,23^ in LMIC. In contrast, only 3.3% of the participants reported cough in our study. Bringing up phlegm was reported by 30% of HIV-negative and 42% of PLHIV in studies conducted in HIC,^18,21,24^ whereas this was reported by only 2% of the participants in our study. Both coughing and bringing up phlegm were reported to happen more in PLHIV than in HIV-negative participants.^18,21,24^ Wheezing and whistling have only been evaluated in two studies from HIC. No difference was found between PLHIV and HIV-negative participants.^24,25^

In contrast, breathlessness was a frequently expressed complaint with 22% of the participants in our study, indicating that they experienced breathlessness. This is in line with the literature, mostly from HIC, reporting that between 1.4% and 42% of the population experience breathlessness. People living with HIV were found to experience breathlessness more often than HIV-negative individuals,^18,25,26^ a finding that we could not confirm in our study.

The following reasons should be considered to understand the low number of respiratory complaints, except for breathlessness, noted among the participants in our study compared to previous studies. Firstly, our study population differed from study populations in HIC. People living with HIV from HIC represent a particular group with specific health risk behaviour, such as men having sex with men (MSM) or intravenous drug users with a higher percentage of smokers than the HIV-negative population.^27^ In our study cohort, PLHIV smoked significantly less than the HIV-negative population (15.2% of PLHIV versus 36.8% of HIV-negative participants, *p* < 0.01). Secondly, with regard to LMIC, most of the studies from the African countries reported that the use of wood fire was still common and responsible for a high burden of respiratory disease and symptoms,^28,29^ whereas in our study household air pollution because of open fires for cooking and heating was hardly present among the participants. Thirdly, heterogeneity in interpretation of definitions may have confounded results among the different studies from HIC and LMIC.

Finally, most of the studies conducted previously in Africa had a low percentage of participants on treatment with ART,^21,30^ whereas 74% of PLHIV in our study were on ART. Uncontrolled HIV infection, and hence inflammation and a high risk of pulmonary infections, would likely result in an increased incidence of respiratory symptoms.

Despite these possible explanations for our findings, it is still particularly surprising that HIV-positive, ART-naïve participants in our study hardly reported respiratory symptoms. A possible explanation could be the fact that the ART-naïve group was still young and had not had the chance to develop symptoms yet. Additionally, they had a median CD4 count of 281 cells/mm^3^ and therefore were still at low risk for opportunistic infections.^31^ This is supported by the low prevalence of a history of tuberculosis in the ART-naïve group compared to the group on first- and second-line ART, which might indicate that ART-naïve participants had not had uncontrolled viremia for a long period of time.

Another unexpected finding of our study was that despite the high burden of respiratory infections in the past in PLHIV, this did not result in a higher frequency of respiratory symptoms except for breathlessness. It may be the case that, given the relatively young age of the population, there was still enough pulmonary reserve capacity to limit complaints in daily life. Clearly, the next step to clarify the relationship between respiratory infections in the past, particularly tuberculosis, and current pulmonary function would be to include lung function testing in the analysis.

Limitations of this study include the recruitment process of the study population which may influence the generalisability of our results. Participants were not randomly sampled from the general HIV-positive population but were recruited from RCTs. However, we think that the participants were representative of people with the same HIV and ART status as they were all recruited from routine local HIV diagnostic services. Furthermore, it is possible that the control group experienced more breathlessness as a result of the high percentage of smokers in this group. This could have reduced the contrast between the HIV-positive and HIV-negative participants.

Lastly, although we tried to define respiratory complaints clearly, there may still be uncertainty around the definition. Future studies should seek to standardise the definition of respiratory symptoms more strictly and it would be helpful if all studies used the same definitions.

In conclusion, the results of this study among a large group of HIV-positive and -negative patients do not support the view that respiratory symptoms are more common among patients with HIV.
