# Erratum: Unexpected low frequency of respiratory symptoms in an HIV-positive urban sub-Saharan population compared to an HIV-negative control group

**DOI:** 10.4102/sajhivmed.v22i1.1180

**Published:** 2021-04-19

**Authors:** Maren Kummerow, Erica J. Shaddock, Kerstin Klipstein-Grobusch, Roos B. Barth, Diederick E. Grobbee, W.D. Francois Venter, Charles Feldman, Alinda Vos

**Affiliations:** 1 Julius Global Health, Julius Center for Health Sciences and Primary Care, University Medical Center Utrecht, Utrecht University, Utrecht, the Netherlands; 2 Division of Pulmonology, Department of Internal Medicine, Charlotte Maxeke Johannesburg Academic Hospital, Johannesburg, South Africa; 3 Faculty of Health Sciences, University of the Witwatersrand, Johannesburg, South Africa; 4 Division of Epidemiology and Biostatistics, School of Public Health, Faculty of Health Sciences, University of the Witwatersrand, Johannesburg, South Africa; 5 Department of Internal Medicine and Infectious Diseases, University Medical Center Utrecht, Utrecht University, Utrecht, the Netherlands; 6 Wits Reproductive Health and HIV Institute, Faculty of Health Sciences, University of the Witwatersrand, Johannesburg, South Africa; 7 Department of Internal Medicine, Faculty of Health Sciences, University of the Witwatersrand, Johannesburg, South Africa

In the version of this article initially published, Kummerow M, Shaddock EJ, Klipstein-Grobusch K, et al. Unexpected low frequency of respiratory symptoms in an HIV-positive urban sub-Saharan population compared to an HIV-negative control group. S Afr J HIV Med. 2019;20(1):a1010. https://doi.org/10.4102/sajhivmed.v20i1.1010, the name of the sixth author was given incorrectly. The correct name should be W.D. Francois Venter instead of Francois D.F. Venter in the ‘Authors’ section.

This correction does not alter the significance of the study findings or the overall interpretation of the study results. The publisher apologises for any inconvenience caused.

